# Using Behavioral Intervention Technologies to Help Low-Income and Latino Smokers Quit: Protocol of a Randomized Controlled Trial

**DOI:** 10.2196/resprot.5355

**Published:** 2016-06-14

**Authors:** Ricardo F Muñoz, Eduardo L Bunge, Alinne Z Barrera, Robert E Wickham, Jessica Lee

**Affiliations:** ^1^ Palo Alto University Palo Alto, CA United States; ^2^ i4health Palo Alto, CA United States

**Keywords:** smoking cessation, Web app, human-centered design, recruitment, dissemination

## Abstract

**Background:**

The Institute for International Internet Interventions for Health at Palo Alto University proposes to develop digital tools specifically to help low-income English- and Spanish-speaking smokers to quit. Individuals from lower-income countries and those with lower social status quit at lower rates than those from high-income countries and those with higher social status.

**Objective:**

We plan to launch a project designed to test whether a mobile-based digital intervention designed with systematic input from low-income English- and Spanish-speaking smokers from a public-sector health care system can significantly improve its acceptability, utilization, and effectiveness.

**Methods:**

Using human-centered development methods, we will involve low-income patients in the design of a Web app/text messaging tool. We will also use their input to improve our recruitment and dissemination strategies. We will iteratively develop versions of the digital interventions informed by our human-centered approach. The project involves three specific aims: (1) human-centered development of an English/Spanish smoking cessation web app, (2) improvement of dissemination strategies, and (3) evaluation of resulting smoking cessation web app. We will develop iterative versions of a digital smoking cessation tool that is highly responsive to the needs and preferences of the users. Input from participants will identify effective ways of reaching and encouraging low-income English- and Spanish-speaking smokers to use the digital smoking cessation interventions to be developed. This information will support ongoing dissemination and implementation efforts beyond the grant period. We will evaluate the effectiveness of the successive versions of the resulting stop smoking Web app by an online randomized controlled trial. Increased effectiveness will be defined as increased utilization of the Web app and higher abstinence rates than those obtained by a baseline usual care Web app.

**Results:**

Recruitment will begin January 2016, the study is intended to be completed by summer 2018, and the results should be available by fall 2019.

**Conclusions:**

This study will provide useful knowledge in developing, testing, and disseminating mobile-based interventions for low-income smokers.

**ClinicalTrial:**

ClinicalTrials.gov NCT02666482; https://clinicaltrials.gov/ct2/show/NCT02666482 (Archived by WebCite at http://www.webcitation.org/6gtcwaT28)

## Introduction

### Background

Smoking is a major health problem. Globally, it is the number one preventable cause of premature death. The World Health Organization estimates as many as 1 billion tobacco-related deaths will occur in this century if smoking trends remain stable [1]. Smoking has a disproportionate impact on low-income populations. Most of the predictable deaths due to smoking are expected to occur in populations from middle- and low-income countries [2]. The state of California has a large proportion of immigrants and descendants of immigrants from such countries, with the largest proportion being from Latin America [3]. As of March 2014, California residents of Latino ethnicity became the largest ethnic group in the state (39% of the total); white residents accounted for 39% of the population [4]. It is estimated that 29% of Californians speak Spanish [5]. Given that Latinos are now the largest ethnic group in California and they are overrepresented in the low-income population of the state, efforts to reach low-income individuals in California must take into account the preferences of Spanish- and English-speaking Latinos.

Low-income populations in the United States have higher smoking rates than high-income populations [6]. Although the overall smoking rate in California is now one of the lowest in the country (14% vs 21%), low-income groups have higher rates. In California, smoking prevalence is 4 times higher among low socioeconomic status (SES) populations than high SES populations (17% vs 4%, respectively) [7]. Safety-net hospitals that serve more low SES patients also reflect disproportionately high smoking rates among their patients. According to Benowitz et al [8], 40% of inpatients surveyed at San Francisco General Hospital, the hospital serving the San Francisco Health Network (SFHN), were active smokers, and 32% of those who denied smoking were found to have had significant exposure. Spanish-speaking groups in California have intermediate rates (13%) [9]; however, Spanish-speaking smokers have lower rates of utilization of smoking cessation aids [10,11]. In a recent paper by our group reviewing 10 years of global online studies [12], we found that nicotine replacement therapies were used by 41.70% of English-speaking smokers but only 20.61% of Spanish-speaking smokers. The nicotine patch was used by 19.50% of English speakers versus 7.70% of Spanish speakers. The same is true for smoking cessation groups: only 2.10% of English speakers and 1.70% of Spanish speakers used them. The lower utilization among this sample of Spanish-speakers could be attributed to limited access to smoking cessation aids in other countries. In the United States, however, access to such aids is widespread, yet utilization remains low among this disproportionally impacted population. A systematic approach that explicitly addresses this well-documented instance of disparities in utilization of smoking cessation tools is called for. We propose to address this issue by systematically involving low-income English- and Spanish-speaking smokers in the development and testing of a new smoking cessation Web app. We intend to eventually disseminate the resulting Web app to low-income smokers in other regions of the country and the world.

### Prior Research and Interventions

Our work on smoking cessation with Spanish speakers began in the 1990s. With support from a grant from the National Cancer Institute (NCI) (Eliseo J. Pérez-Stable, principal investigator), we attempted to carry out a traditional smoking cessation study involving face-to-face group interventions. Initial attempts to engage smokers in traditional face-to-face cessation groups were not successful. However, an alternative strategy of using surface mail was far more successful in reaching smokers. We found that our smoking cessation guide (*Guía Para Dejar de Fumar*) yielded 11% quit rates at 3 months, and adding a brief mood management intervention doubled the quit rate to 23% [13]. Our experience with this study highlighted the importance of finding alternate means of reaching Latino smokers and providing them with resources that could be effective when used at home and could be offered to the community at no charge. Seeing the possibilities for reaching people at home using the Internet, we submitted a grant to California’s Tobacco-Related Disease Research Program (TRDRP) in 1997 proposing developing and testing an online intervention to help Spanish- and English-speaking smokers in California stop smoking. Our team has received three TRDRP grants which have resulted in 19 publications to date. Below we summarize our studies and list several lessons learned from our experience. The current grant proposal is intended to implement the logical next steps in our research program based on what we have learned thus far.

Our studies have yielded proof of concept that efficacy of Internet interventions for smoking cessation are higher than those reported for placebo patches (9% at 6 months [14]) and can approach those reported for the nicotine patch (6-month quit rates of 22% [14]) and smoking cessation groups (6-month quit rates of 27% for American Lung Association groups and 24% for American Cancer Society groups [15]). Our most robust missing=smoking abstinence data (a conservative convention in which all participants with missing data at any follow-up are presumed to be smoking which allows us to include all randomized participants in outcome analyses, similar to intention-to-treat approaches) at 12 months are 20% for Spanish speakers and 21% for English speakers for smokers with mean Fagerström scores of 5.2 [16], similar to those scores found in face-to-face smoking cessation trials by Hall et al [17].

One of our surface mail interventions was labeled *Tomando Control de su Vida* (*Taking Control of Your Life*) which led to the TC designation for the series of studies that followed. TC1 was the NCI-funded randomized controlled trial (RCT) conducted via surface mail (entirely in Spanish) in which we found that a smoking cessation guide together with a mood management intervention significantly improved quit rates [13]. In order to increase the number of smokers we could potentially reach, we obtained TRDRP funding to adapt our materials into an automated self-help Internet intervention, the San Francisco Stop Smoking website. This site was used in several online randomized controlled and participant preference trials (TC2, TC3, and TC4).

The TC2 studies [18] involved over 4000 smokers from 74 countries and examined outcome (self-reported 7-day abstinence) and mechanisms related to outcome (the impact of major depressive episodes on the likelihood of quitting). It included four substudies. Substudies 1 and 2 evaluated the online version of the smoking cessation guide, and substudies 3 and 4 were randomized trials comparing the guide plus ITEMs (individually timed educational messages) to the guide plus ITEMs and a mood management course. At 6 months, self-reported 7-day abstinence rates using the missing=smoking convention varied from 5.60% (39/702, study 2) to 25.56% (38/146, study 4 ) in the four substudies. The guide plus ITEMs condition tended to have higher quit rates. The best missing=smoking abstinence rate (28.10%, 41/146; study 4) at 3 months was found in a sample of Spanish-speaking smokers.

The TC3 studies [18,19] consisted of a totally automated self-help smoking cessation site programmed to randomly assign participants who signed consent to one of four conditions. Each condition added new elements: condition 1 consisted of the static smoking cessation guide, condition 2 consisted of the guide plus email reminders to return to the site, condition 3 added cognitive behavioral mood management lessons, and condition 4 added a virtual group (an asynchronous bulletin board). The first phase of the trial included the first 1000 participants recruited (from 68 countries); 500 Spanish-speaking and 500 English-speaking adult Internet users were randomized to the four study conditions and, in order to reduce attrition at follow-up, were contacted by phone if they did not provide 1-, 3-, 6-, and 12-month follow-up data online. With the addition of phone follow-ups, we obtained follow-up rates of 73.50% (735/1000), 66.10% (661/1000), 57.30% (573/1000), and 69.20% (692/1000) at 1-, 3-, 6-, and 12-month follow-ups, respectively. There were no significant differences among the four conditions. However, the overall 12-month 7-day missing=smoking abstinence rates were 20.20% (101/500) for Spanish speakers and 21.00% (105/500) for English speakers.

After recruiting the first 1000 participants, we maintained the TC3 website online, and we conducted a totally automated RCT (that is, we did not follow participants via the phone) in which 16,430 participants from 165 countries were recruited and followed entirely via automated emails [19]. The totally automated trial had, as expected, a much greater attrition rate than the initial TC3 trial. For month 1 follow-up, 39.95% (6563/16,430) of participants provided data. This number was reduced to 30.38% (4992/16,430), 23.21% (3813/16,430), and 21.95% (3606/16,430) for follow-ups at months 3, 6, and 12, respectively. These numbers were comparable with those obtained in the earlier TC3 study, in which 36.37% (4952/13,617), 26.92% (3666/13,617), 20.77% (2828/13,617), and 18.98% (2585/13,617) of participants who never received any live follow-up returned at months 1, 3, 6, and 12, respectively. The self-reported 7-day observed quit rates ranged from 36.18% (2239/6189) at 1 month to 41.34% (1361/3292) at 12 months. The 7-day missing=smoking abstinence rates ranged from 13.63% (2239/16,430) at 1 month to 8.28% (1361/16,430) at 12 months.

In the TC4 studies [20] we morphed the site into a participant preference site. The Spanish/English San Francisco Stop Smoking site was designed to be open. Specifically, any Spanish- or English-speaking smoker 18 years of age or older anywhere in the world could enter the study and select (rather than be randomized to) any of the intervention elements tested in our previous studies. Our intent was to demonstrate that Internet interventions tested in RCTs can be readily adapted to serve as universal health care resources. During the first year of the study, 94,158 individuals from 152 countries and territories visited the site. The participant preference design allowed us to examine quit rates obtained when users could choose from all elements tested in previous RCTs. Participants were able to personalize the site by choosing among 9 site elements (e.g., stop smoking guide, reminder emails, journal, mood management intervention, virtual group). Results from the first year of recruitment yielded higher observed quit rates (odds ratio 1.30) than the previous RCT (TC3) when controlled for individual demographic and smoking characteristics [20]. Of note, the utility of this version of the site as an adjunct to traditional health care has been tested by an independent research group. Gallego and colleagues [21] used the San Francisco Stop Smoking site with smokers at a clinic in Spain as an adjunct to standard pharmacological treatment and report posttreatment abstinence rates of 78.13% (25/32) and 1-year follow-up abstinence rates of 53.13% (17/32). This study suggests that our digital tools can serve as adjuncts to usual primary care, motivating us to involve the SFHN as described below.

Our latest study was on a new sample of participants who were recruited for 18 additional months to the San Francisco Stop Smoking site. A total of 164,182 individuals from 168 countries visited the site during this period: 27,163 were screened for eligibility; 9348 signed consent; 7407 completed the baseline survey; and 1431, 901, 595, and 318 left 1-, 3-, 6-, and 12-month data, respectively. Observed quit rates were 39.20% (561/1431), 43.51% (392/901), 45.71% (272/595), and 50.31% (160/318), respectively. The TC4 studies show that Internet interventions can yield quit rates similar to those found in earlier RCTs and can be disseminated to literally thousands of users across the world with minimal additional costs. We have begun thinking of them as MOOIs (massive open online interventions) [22], similar to the well-known MOOCs (massive open online courses). The current proposal will help us design a MOOI with greater reach than our previous website by incorporating preferences that take into account demographic trends in technology access and use [23]. Specifically, we will recruit smokers with lower income and educational levels to help us design a Web app that is more accessible and acceptable to them.

### Clinical Problem

We have argued that Internet interventions can help reduce health disparities by reaching those who do not have access to smoking cessation aids. Concerns have been raised that, although this approach may be increasing the reach of evidence-based smoking cessation interventions across large segments of the population and thus reducing health disparities across countries, digital interventions may actually increase health disparities. The reason for this concern is that more highly educated people may be more likely to use these interventions and perhaps more likely to benefit from them. To address this concern, we have compared quit rates for participants with differential socioeconomic standing as well as for participants from countries with different gross domestic products. We tested whether smokers in richer countries and smokers higher in self-reported SES are more likely to quit. Our results show that, indeed, our Internet interventions preferentially benefit individuals from countries with higher gross domestic products and/or with higher individual socioeconomic standing [24]. This may be due to reasons other than the availability of technology because all participants had access to the online intervention. It may be that the ways in which we have designed the interventions do not take into account the characteristics of smokers with lower incomes and lower educational levels. These findings are the motivation for the current study. We have received TRDRP funding to revise our smoking cessation digital tools so that they address the unique interests and needs of low-income and Latino smokers in California with the hope that the modifications made will generalize to other smokers from similar socioeconomic levels. We conceptualize the SFHN, described in the next paragraph, as serving as a “magnifying glass” that will allow us to learn how to best serve this population with our digital tools so that when we make the tools available worldwide, the range of smokers we will be able to reach and be effective with will be greater.

The SFHN is the city’s only complete care system. It consists of 14 primary care clinics throughout the city as well as San Francisco General and Laguna Honda hospitals. Between July 1, 2013, and June 30, 2014, the SFHN served 72,379 unduplicated patients who had 354,445 outpatient visits. Almost all were covered by Medi-Cal and/or Medicare (251,616/354,4445, 71.00%) or were uninsured (101,466/354,445, 28.60%). Only 60.10% (43,460/72,379) reported English as their primary language; 22.01% (15,905/72,379) reported Spanish as theirs. The next largest language group was Cantonese (8042/72,357, 11.10% ) followed by an additional 37 language groups, all at 1.20% (853/72,357) or less. Latinos are the largest ethnic group being served, at 32.60% (23,575/72,357) of the total, followed by Asians (25.20%, 18,206/72,357), whites (19.01%, 13,721/72,357), African Americans (18.30%, 13,214/72,357), and other groups accounting for less than 2.20% (1585/72,357) each. Slightly more than half of the patients are women (53.10%, 38,398/72,357). Those between the ages of 18 and 64 years (the group most likely to participate in our studies) compose 69.40% (50,355/72,357) of the population. Smoking rates are likely higher than for the general San Francisco population (13%), but rates are not available for the SFHN as a whole. Benowitz et al [8] have found that 40% of San Francisco General Hospital inpatients were active smokers, and of those who denied smoking, an additional 32% were found to have significant exposure to nicotine. Thus the smoking rate in primary care patients is likely higher than 13% but less than 40%. Assuming a 20% smoking rate, there are likely to be 8247 smokers who are aged 18 to 64 years and speak English or Spanish.

Estimates of how many SFHN patients use digital technology stem from a published report by Schickedanz et al [25] who surveyed 416 patients in waiting rooms of SFHN clinics and report that 71% were interested in using electronic communication with health care providers and 19% were already doing so. The survey was conducted in English, Spanish, Cantonese, and Mandarin; 87% (52/60) of Spanish speakers were interested in using email to communicate with their health care provider compared to 77% (164/213) of English speakers, 38% (23/61) of Cantonese speakers, and 75% (38/51) other. It appears, then, that interest in digital tools is high even in this low-income population. We have chosen to recruit SFHN patients who own smartphones because we believe that by 2018 most will do so and we want our intervention to be relevant for that time.

We propose to recruit the following number of SFHN smokers who use smartphones: face-to-face field studies (ethnographies and usability tests), 30 in year 1 and 30 in year 2 and online studies, 50 in year 1, 100 in year 2, and 200 in year 3. We believe recruiting 410 out of more than 8000 smokers over a 3-year period is feasible.

We will also be doing open recruitment of smokers who own smartphones throughout California at the following rate: 100 in year 1, 200 in year 2, and 800 in year 3. This effort will provide a large enough sample to carry out adequately powered outcome studies as described in the Methods section.

The proposed study is based on the premise that we cannot presume to know what would be best for our intended users without engaging them. To build a smoking cessation intervention that will be used and be effective with low-income smokers aged 18 years and older, including Spanish-speaking smokers, we must build a deep understanding of their needs and values.

We propose to carry out a research study inspired by the “rapid, responsive, relevant (R3) research” approach described by Riley and colleagues [26]. The R3 approach urges researchers to consider shifting away from a narrowly focused and long-term RCT approach and to instead use techniques that reflect and integrate stakeholder needs, use innovative designs and methods for evaluation, and emphasize widespread immediate implementation. A successful R3 research approach is scientifically rigorous and a good fit for addressing mental health disparities in underserved communities such as low-income Latino smokers, where uptake of empirically supported interventions has failed or is not relevant to the cultural or technological characteristics of the target population.

### Lessons Learned From a 16-Year Online Research Program

There are more than 1.1 billion smokers worldwide [27]. We will never train enough health care providers to administer adequate health care for all who need it if we continue our reliance on consumable interventions (ie, interventions that once used cannot be used again). For example, the nicotine patch can only be used once, and the time spent counseling a patient to quit smoking cannot be used again to help another patient. To reduce health disparities, we need interventions which can be used again and again without losing their therapeutic power, can reach people even if local health care systems cannot or will not provide them with needed health care, and can be shared widely without taking resources away from the populations for whom the interventions were developed.

A major advantage of fully automated self-help digital interventions is their low cost. Bringing a medication to market costs hundreds of millions of dollars [28], while developing a digital intervention may cost as little as a few thousand dollars for a simple site. Costs of testing a site in RCTs may run in the hundreds of thousands of dollars but generally cost less per trial participant than face-to-face interventions. The differences in per-person costs of administering the intervention are even more impressive: a dose of a medication has a minimal cost even when bought in large quantities, but the marginal cost of providing a self-help automated Internet intervention to one additional user eventually approaches zero. The cost of hosting the site remains steady once the server has made the site available to say, 50,000 users, and the cost of serving one more user becomes negligible. This makes it possible to share the site with people worldwide. We have shown that, with TRDRP funding, we have been able to help California smokers and, at no additional cost, smokers all over the world.

Our research group has shown that unguided interventions focused on depression have significantly reduced depressive symptoms [29]. Our TC studies were all conducted as self-help automated interventions. Thus, we propose to continue to focus on automated interventions, moving from desktop- or laptop-accessible websites to mobile devices (mostly smartphones) which can access Web apps and text messaging throughout the smoker’s day. It is true that digital interventions do not need to be fully automated. For example, Internet interventions can employ staff to provide support, information, and encouragement to users. However, such guided interventions involve staff time, which places them in the category of consumable interventions. Such interventions are generally limited to the geographical area that provides the salary for the support staff; offices, equipment, and communication tools; and organizational infrastructure to provide the guidance. Once grant funding ends, the guided intervention has to end. By focusing our work on self-help automated interventions, we increase the likelihood that we will be able to keep these interventions available to people beyond the grant period and beyond the geographical area covered by the grant.

When the principal investigator first began to work at San Francisco General Hospital in 1977, he believed that, given ten years of recruiting and training new Spanish-speaking health care providers, we would be able to hire enough Spanish speakers to meet the demand for services in our hospital. More than 39 years later, we are still having difficulty recruiting Spanish-speaking providers. We are often forced to either not provide Spanish speakers needed services or to use interpreters to communicate with them. Smoking cessation groups in Spanish are hard to maintain. Our group has made the development of interventions in both English and Spanish (and, whenever possible, in other languages) one of its major priorities. Thus, this proposal focuses on low-income Latino smokers, most of whom speak Spanish and have low English proficiency.

When we began our work with online smoking cessation tools, we used skeuomorphic designs. A skeuomorph is a physical ornament or design on an object made to resemble another material or technique. The lights of an electric candelabra are fashioned in the shape of traditional candles, but the candle shape is not necessary to provide light. The shutter sound on cameras built into mobile devices and the floppy disk icons used on computers to save documents are, likewise, skeuomorphs. We included an 8-session mood management intervention modeled after face-to-face clinical appointments, and few people actually completed the sessions. In this proposal, we are attempting to be mindful of not limiting our designs using skeuomorphic thinking [30]. We propose to observe how digital tools are used by the population we are choosing to serve in their day-to-day lives and design our interventions in ways that are likely to fit the behavioral patterns we observe.

In a recent paper on rapid, responsive, relevant research [26], Riley and colleagues point out that a National Institutes of Health research grant designed in 2005 and awarded in 2006 would miss at least six important consumer technology advancements between its award and its publication in 2012, including the Wii, iPhone, iPad, and Siri. In our earlier projects, we designed a website, kept it as originally designed for the length of the RCT, and continued to use it beyond the grant period. There are advantages to that strategy, of which the most obvious is cost. Keeping the site originally developed with TRDRP funding awarded in 1998 still operational in 2014 was a rather remarkable example of good use of funds. However, the site eventually looked outdated, and technology is moving at a much faster rate than it did in 1998. It is important that our digital interventions be much more flexible than they were in the late 1990s. We propose an agile development process that will allow us to shape our intervention so that it is not frozen at the start of the grant period and can become increasingly responsive to the needs and preferences we discover in our population of interest. We intend for the Web app we develop to have a current look and feel when the grant ends in 2018. Specifically, we will engage stakeholders (low-income and Latino smokers and their health care providers in a large urban public-sector health network) in the development of the smoking cessation interventions with the goal of increasing follow-up retention and disseminating results so that other communities can take advantage of our interventions.

### Objectives

#### Aim 1: Human-Centered Development of an English/Spanish Smoking Cessation Web App

We will develop iterative versions of a digital smoking cessation tool (a Web app with text messaging components) that is highly responsive to the needs and preferences of low-income English- and Spanish-speaking smokers. Development will take place with systematic input from patients who are part of the SFHN, which serves 70,000 mostly low-income individuals.

#### Aim 2: Improvement of Dissemination Strategies

Input from SFHN patients will identify effective ways of reaching and encouraging low-income English- and Spanish-speaking smokers to use the digital smoking cessation interventions that are developed. This information will support ongoing dissemination and implementation efforts beyond the grant period.

#### Aim 3: Evaluation of Resulting Smoking Cessation Web App

We will evaluate the effectiveness of the successive versions of the resulting stop smoking Web app by recruiting smokers at two levels—within the SFHN and throughout the state of California—culminating in an online RCT. Increased effectiveness will be defined as increased use of the Web app and higher abstinence rates than those obtained by a baseline (usual care) Web app.

#### Hypotheses

H1: Utilization of the final smoking cessation Web app (number of screens viewed, time spent using app, and amount of data entry) will be significantly higher than that of the baseline Web app.

H2: Recruitment based on dissemination strategies derived from ethnographic interviews will reach a significantly higher number of participants for successive online studies.

H3: Abstinence rates (7-day self-reported abstinence) will be significantly greater for the final Web app than for the baseline Web app.

## Methods

### Conceptual Foundations of the Research Design

#### Design Thinking and Health Care

Design thinking is a user-centered approach to innovation that considers and integrates the needs of people within the possibilities and reach of technology [31,32]. Design thinking is not just for designers—it is a mindset and process of problem solving that aims to identify the core tenets that drive a specific behavior. This knowledge is then used to solve complex problems and find desirable solutions. The emerging trend of applying user-centered design to health care problems has generated promising results [33]. Skinner et al [34] employed a user-centric approach in developing a youth smoking prevention and cessation website and demonstrated in a large-scale RCT that the intervention yielded positive effects. The Center for Innovation at the Mayo Clinic uses design thinking as a core process in creative problem solving that goes beyond process analysis and quality improvement [35]. The Innovation Consultancy at Kaiser Permanente used design thinking to improve the quality of patient care during nursing shift changes [36]. The Venice Family Clinic, a community health care center in California, used the design thinking process to redesign their clinic and patient experience for low-income families [37].

#### Design Thinking Processes

There are many variations of user-centered design processes, but they all uphold the same core tenets of being human-centered, prototype-centric, and iterative. The design thinking model presented by Kembel [38], notably different for its explicit treatment of empathy, is most relevant for the goals of this study. In this model, empathy arises from a deep understanding of the stakeholders and their needs and requires an anthropological approach to understanding users and their environments. In this study, we will leverage the design thinking process presented by Kembel to create and distribute a digital intervention for English- and Spanish-speaking low-income smokers. The model consists of a 5-step cyclic process.

- Empathize: fully understand the experience of the intended user via observation, interaction, and immersion in their experiences. This entails understanding their lifestyle, physical and emotional needs, how and why they do things, and what is meaningful to them as it relates to their smoking behavior. The goal is to uncover insights that will guide design and distribution of our digital intervention.
- Define: process and synthesize data collected from the Empathize step to outline connections and patterns of our target users. The goal is to develop a defined, meaningful, actionable problem statement that will provide focus when developing and evaluating proposed solutions.
- Ideate: generate a variety of possible solutions to provide source material for building prototypes. It is critical during this step to defer judgment and push for a wide range of ideas, allowing us to think beyond obvious existing solutions. The best solution is eventually determined during the prototyping and testing steps. We propose to systematically involve smokers and their health care providers in this step.
- Prototype: transform ideas into physical forms so users can interact with them. Initial low-fidelity prototypes (eg, sketch drawings, storyboards) are quick and inexpensive ways for the team to learn how well a solution aligns with the user needs. Each iteration moves the team closer to a final, user-defined solution.
- Test: observe and elicit feedback to learn more about the user, refine prototypes, and gain clarity on how well the solution addresses the problem statement.

### Research Design and Methods

#### Framework

##### Human-Centered Development

We will develop iterative versions of digital interventions for smoking cessation in Spanish and English that are highly responsive to the needs and preferences of low-income and Latino smokers. Development will take place with user input from patients who are part of the SFHN. We will use the 5-step cyclic design thinking process described above to understand low-income smokers and iteratively develop and refine an effective digital smoking cessation Web app that they will use.

##### Dissemination Strategies

We will include in the ethnographic semistructured interviews questions regarding how the users found out about Web apps installed on their phones, how they use text messaging, and which digital smoking cessation tools they have used the most (if any). This information will guide the development of our recruitment and dissemination strategies both at the SFHN and at the state level.

##### Evaluation of the San Francisco Stop Smoking Web App

We will develop a baseline Web app and 3 successive versions of the user-centered Web app, the last of which will be evaluated in an RCT.

#### Initial Ethnography (Empathy Step)

##### Recruitment

**Figure 1:**
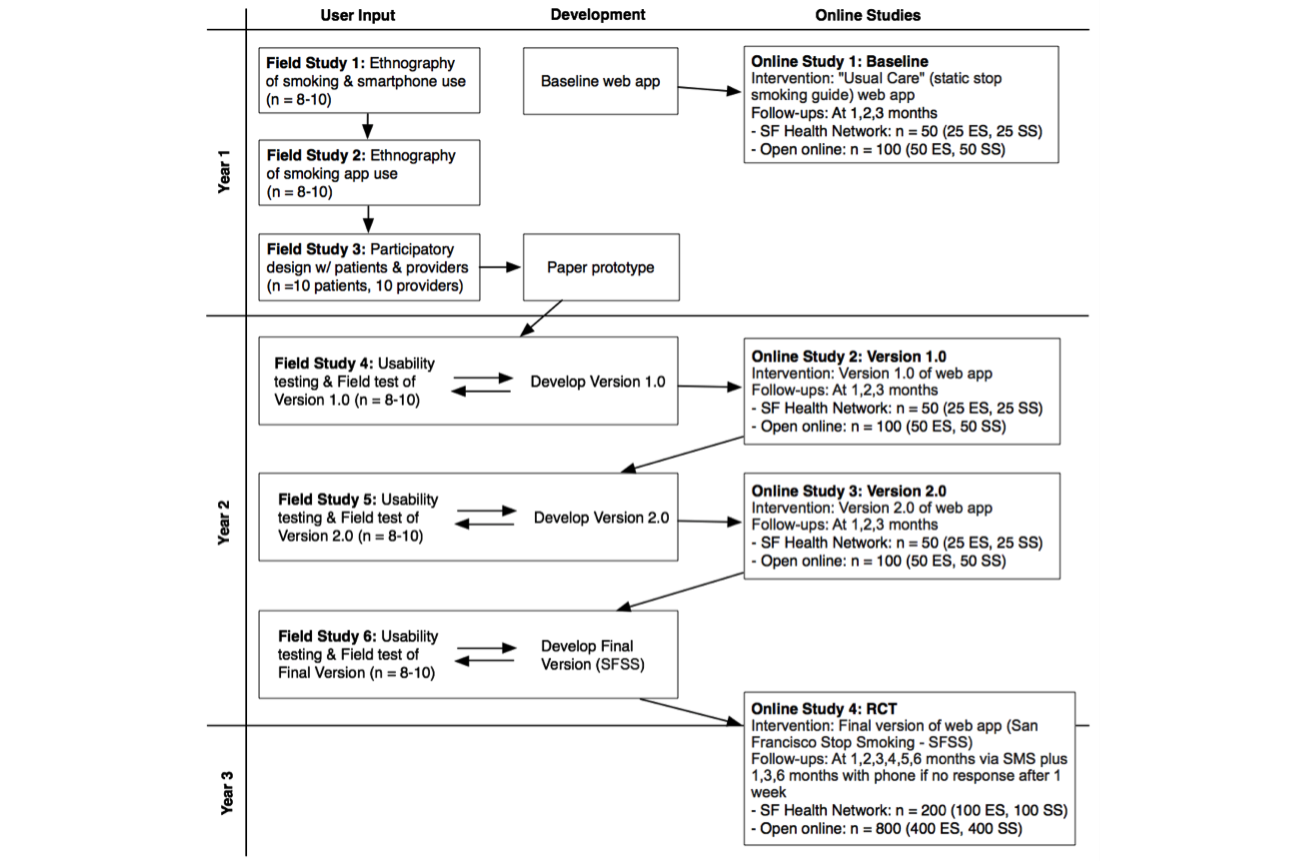
Flow of research study.

We will recruit 60 low-income participants aged 18 years or older who are members of the SFHN, use smartphones, and have been advised by their care providers to quit smoking or have tried to quit smoking at least once. They will be divided into four target groups (15 English-speaking men, 15 English-speaking women, 15 Spanish-speaking men, 15 Spanish-speaking women). These 60 participants will be asked to sign consent to participate in the shadowing process and short or in-depth interviews conducted by research assistants. We will also recruit 10 health care providers from the SFHN (eg, smoking cessation counselors, nurses, physicians) to contribute to the Ideate step by taking part in a prototyping exercise (see Figure 1, Field Study 3).

##### Methods

To ensure that our solution is based on a solid foundation, we will invest significant efforts during the initial Empathy step to understand our users. We will employ ethnographic methods to build a deep understanding of low-income English- and Spanish-speaking smokers. Research assistants will receive ethnography training (manuals and mock interviews) prior to going out into the field. We will conduct semistructured interviews pertaining to our themes of interest. Short interviews are hour-long semistructured interviews where research assistants will recruit patients from SFHN clinic waiting rooms to ask a set of questions related to their lifestyle and smoking habits. In-depth interviews will include a short interview plus a follow-up interview after a quit attempt and shadowing period of 3 hours in a natural location for the participant. Relevant themes include behaviors related to smoking, technology usage, personal lifestyle and social circles, how they get information (informing dissemination strategies), and personal values and motivations. All interviews will be audiorecorded and sent to third-party services for transcription.

##### Outputs and Qualitative Analysis

Our ethnographic methods will yield a large amount of transcribed material, screenshots, and other photographs showing the use of apps. Our qualitative analysis will involve developing and refining our themes, identifying quotes from participants that support each theme, and developing a coding system that will help us identify behavior patterns and draw conclusions that will inform the next step in the design process.

#### Implementing the User-Centered Design

##### Recruitment

SFHN patients will be recruited by research assistants at outpatient clinics. We will seek patients who own smartphones and intend to quit smoking in the next 3 months who either have been advised to quit smoking by their care provider or have tried to quit smoking at least once. Patients will be paid $25 per hour for their time for interviews as described in face-to-face studies below.

##### Procedures

User-centered design, development, and testing will be carried out as shown on Figure 1, with two interconnected processes running in parallel: face-to-face user input from SFHN smokers via ethnographic and field test methods and online studies that will include samples recruited from the SFHN and open online recruitment. In all studies, half of the participants will be English-speaking and half Spanish-speaking.

#### Field Studies

##### Recruitment

For every field study the eligibility criteria are 18 years of age and older, current smoker (any number of cigarettes per day), and English or Spanish speaker. We plan to recruit low-income participants (ie, those who have Medicare and/or Medi-Cal, the California state equivalent, or are uninsured). Six face-to-face field studies are planned.

##### Year 1

###### Field Study 1: Ethnographic Study of Naturalistic Smoking, Smoking Cessation Attempts, and Smartphone Use

We will use shadowing methods (observing smokers’ actual behavior in their natural environment) plus semistructured interviews to learn how smokers think about their smoking behavior, how they attempt to quit, and how they use their smartphones (ie, how they find, download, install, and begin using apps).

###### Field Study 2: Ethnographic Study of Smoking Cessation App Use

We will recruit SFHN patients who have installed at least one smoking cessation app on their smartphones. We will use shadowing and semistructured interviews to study the use of smoking cessation apps participants have installed on their own. We will ask participants to install the baseline version of our Web app and use it for one week, scheduling follow-up interviews to learn about use patterns and reactions to the baseline Web app.

###### Field Study 3: Participatory Design Field Test

We will schedule two meetings, one in English and one in Spanish, with 5 SFHN smokers each, with the purpose of jointly working on the design of an ideal app to help them stop smoking. Groups will be facilitated by research staff familiar with Web apps to keep the group focused on the intended product. The result will be a paper prototype and sample screens developed on tablets to ensure that we are accurately rendering the patients’ suggestions. In addition, we will schedule two meetings, one in English and one in Spanish, with 5 SFHN providers each (smoking cessation counselors, nurses, physicians), using the same procedure. The results of the four meetings will be shared with the development team as part of the creation of version 1.0.

##### Year 2

###### Field Study 4: Usability Field Testing for Version 1.0

Once the developers begin the design process, we will engage in iterative usability testing with 4 or 5 SFHN English speakers and 4 or 5 Spanish speakers. Their input will be incorporated in version 1.0, which will be subjected to limited online testing (Online Study 2).

###### Field Study 5: Usability Field Testing for Version 2.0

We will engage in iterative usability testing as version 2.0 is being developed to ensure that SFHN smokers have input into the new version, which will then be subjected to limited online testing (Online Study 3).

###### Field Study 6: Usability Field Testing for the Final Version of the San Francisco Stop Smoking Web App

We will carry out iterative usability testing of the final version to ensure that SFHN smokers have input. The final version will be tested in an RCT.

#### Online Studies

##### Online Study 1: Baseline (Usual Care) Web App

A Web app will be developed to test the data-gathering infrastructure for the subsequent apps and generate baseline information on four variables: recruitment rates, utilization of the app, follow-up completion rates, and quit rates. Usual care is described in the Intervention section. We will engage in focused recruitment of smokers from the SFHN (up to 25 English speakers and 25 Spanish speakers) as well as open online recruitment with California as our main target (up to 50 English speakers and 50 Spanish speakers). We will implement follow-up interviews to examine quit rates at 1, 2, and 3 months after enrollment in the study. We will use 3 text messages (identified as coming from the Stop Smoking Study) 2 days apart. The first question will ask “Have you smoked 1 or more cigarettes in the last seven days?” A no response will be interpreted as 7-day point prevalence abstinence. Additional questions will address 30-day abstinence, number of cigarettes smoked daily if not abstinent, and use of other smoking cessation tools.

##### Online Study 2: Version 1.0

We will implement the same procedure as Online Study 1. We will obtain suggestions from our field test participants as to how to increase recruitment rates, for example, by modifying the wording in our social media announcements, the themes we touch upon in our radio and television interviews, the flavor of our public service announcements, word-of-mouth recruitment, the use of text messages to encourage enrollment, the wording of our search engine ads, and so on. We will use “tokens,” as described below, to identify how the participant heard about the Web app. We will examine which of these sources yield the greatest increases in recruitment. We will also track overall recruitment, as measured by the speed with which we meet our recruitment goals.

##### Online Study 3: Version 2.0

We will repeat the procedure as in Online Studies 1 and 2.

##### Online Study 4: Randomized Controlled Trial

We will carry out an RCT comparing the final version of the San Francisco Stop Smoking Web App with the baseline Web app. The RCT will be powered to detect differences in utilization rates and 7-day point prevalence quit rates, as described in the Data Analysis section. We will carry out open online recruitment, with a target of 800 smokers (400 English speakers and 400 Spanish speakers), as well as focused SFHN recruitment, with a target of 200 smokers (100 English speakers and 100 Spanish speakers).

Open recruitment will focus on California smokers. We will target English- and Spanish-speaking smokers searching for information on smoking cessation online using social media recruitment efforts, appearances on radio and television shows (especially those likely to be watched by Latinos), and a limited amount of search engine ads. To target the latter, we will use information reported by Graham and others [39] in terms of the most cost-effective online sites to recruit Latinos. We will focus our recruitment advertising resources on areas of California that have large proportions of Latinos in order to oversample Spanish-speaking smokers.

SFHN recruitment involves providing fliers for patients in waiting rooms at the San Francisco General Hospital clinics. Waiting room staff will also be given the fliers so they can inform smoking patients about the study. Primary care physicians will be provided with Post-It–like prescription pads which will have information regarding how to obtain the smoking cessation Web app. We will provide tokens (code numbers) to be entered when the Web app is installed that will indicate the source of the referral in order to determine whether physician recommendations to use the Web app result in greater utilization and higher quit rates than standard fliers. These tokens will also distinguish SFHN users from users recruited via open online recruitment.

#### Digital Interventions

##### Baseline (Usual Care) Web App

Online Study 1 will test the data gathering aspects of the proposed Web app using a baseline usual care intervention consisting of a static smoking cessation guide, *Guía Para Dejar de Fumar*, tested in printed form in the Muñoz et al study [13]. The print version of the guide yielded an 11% quit rate at 3 months. We will upload the content of the guide to the baseline app, and it will serve to estimate baseline utilization and quit rates.

##### The San Francisco Stop Smoking Web App

This Web app will be developed with input from the SFHN population. Thus, we are not able to provide a concrete description of the app at this time. However, our work to date has been guided by social learning/social cognitive theory and uses cognitive behavioral principles that will guide our thinking. Based on our experience with Web-based apps, we have found a number of features are valued by users. Users want information provided in a number of ways (text, graphics, audio, video, *telenovela*-type stories). Goal setting is important, so we will be looking for ideas for interactive goal-setting tools. Notifications are useful for bringing people back to the site or Web app, and we will ask our informants if they prefer them via text messaging or triggered by the app itself. We know that small behavior change steps can be encouraged using reinforcement, so we will be on the lookout for gaming ideas such as winning points and earning badges. We will ask for specific skills that our informants see as most useful (eg, saying no when offered cigarettes) and teach these skills using models. We will also consider social interaction tools (eg, smoking cessation buddies). However, the specific instantiation of these principles will stem from the participatory design process as defined by input from SFHN patients and health care providers. The exact structure of the intervention will be guided by participant input. Recent reports suggest using a hierarchical taxonomy to describe the active components of behavioral change intervention protocols [40]. We anticipate that active components of the intervention will include goal setting (behavior and outcome), self-monitoring, social support (practical and emotional), prompts/cues and cue signaling rewards, and behavioral practice/rehearsal.

#### Measures

##### Field Studies

User-centered design and usability testing aims to determine whether people can use the tools and features (ease of use) and how they like using them (usefulness). These questions will be addressed using a mix of qualitative (semistructured interview) and quantitative (satisfaction, acceptability, and perceived usefulness ratings) methods. Furthermore, during usability testing participants will be audio- and videotaped while interacting with the tools and features. Participants will report their overall satisfaction, as well as satisfaction with the design, content, functionality, and features. Participants will report what they liked best and least about the tools and features as well as strengths and weaknesses of the tools. Participants will rate the perceived usefulness of each aspect and the acceptability of using the program in the future.

##### Online Studies

To reduce participant burden, we will use reduced versions of the baseline and follow-up questionnaires we used in our earlier website-centered studies. Being asked to respond to long surveys on a mobile device is likely to result in high dropout rates. Therefore, we propose the following:

- Eligibility: age, current smoker, speak English or Spanish.
- Baseline (obtained on the Web app when the smokers installs the app and gives consent to enter the study): demographics (age, sex, race, ethnicity, marital status, employment status, income, and educational level), smoking history (age first cigarette, age regular smoker, cigarettes per day, quit attempts, and methods used to quit), the Fagerström Test for Nicotine Dependence (6 items) [41], and rating of quit confidence.
- Follow-ups (brief and obtained using text messaging to increase completion rates): smoking status (7-day and 30-day point prevalence abstinence rates, operationalized as a “no” response to “Have you smoked 1 or more cigarettes in the last seven days?” and, if “no” to the previous question, “Have you smoked 1 or more cigarettes in the last 30 days?”); number of cigarettes per day smoked in the last week (if still smoking); and rating of confidence that the user will remain quit (if not smoking) or be able to quit in the next 30 days (if smoking).

#### Data analysis

##### Ethnographic and Field Testing Phases

Mixed-methods data analysis will incorporate qualitative data from patient and therapist feedback; quantitative data from the satisfaction, acceptability, and usefulness ratings; and audio recordings from the field tests. Although quantitative ratings can highlight possible usability concerns, qualitative data provide answers to why those features might cause problems in ways that can guide development. Qualitative data will be interpreted following a grounded theory approach in which results from the semistructured interview will be analyzed using a series of codes to determine patterns in topics related to patients' and therapists' needs, concerns, and impressions of the prototype. Grounded theory was selected because it is a useful methodology for determining common topics within qualitative data to inform future practices and research. For the usability phase, we believe the users will raise unanticipated concerns and needs and thus we selected an approach that is flexible. Mean values of ratings from individual tools and features will be computed to identify those that require further refinement. Any tool or feature that is given the lowest value on a quantitative rating scale will be flagged for more intensive review in the video recordings and screen captures. Audio recordings and screen captures will address ease of use and be coded to determine three types of errors: navigation errors refer to instances when users cannot locate a function or have difficulty with aspects of the screen flow, content errors refer to instances of problems due to labeling or information presented, and usage errors refer to improper tool use or data field entry. Qualitative and quantitative data will be integrated through linking categories identified using the grounded theory approach with ratings on tools and features. Tools and features must score a mean value in the satisfactory range to be included in the next version. We will consider developing additional tools and features for other categories identified by users. Consistent with principles of constant comparative analysis, the principal investigator will make final decisions regarding tools and features.

##### Online Studies 1, 2, and 3

These studies are intended to provide continuous quality improvement data as a baseline smoking cessation intervention (a static stop smoking guide) moves through two iteratively designed versions of a user-centered Web app. The final version of the Web app will be evaluated in comparison to the baseline app. We will examine successive changes in recruitment rates, utilization of the app, follow-up rates, and quit rates. We will use observed changes or lack thereof to pinpoint areas that need improvement; this will help prioritize changes in recruitment media and messages, Web app functionality, text messaging issues, and elements of the Web app likely to increase quit rates.

##### Online Study 4

Participants will be randomly assigned to use either the baseline app or the final version of the San Francisco Stop Smoking Web app. We will focus our analysis on quit rates and use of the app. Latency (time spent) on content pages will be compared across the two versions of the app using independent samples *t* tests. Based on a Cohen designation of a small effect size for group mean comparisons of 0.2, a priori power analyses revealed a necessary sample size of N=800 (400 per version) to achieve a power of .80. Quit rates (smoking cessation status at 7- and 30-days) will be evaluated at 1, 3, and 6 months following registration, with individuals lost to follow-up treated as still smoking. To examine quit rate as a function of website version and demographic characteristics, logistic regression analyses will be conducted with quit status as the criterion variable; website version as the focal predictor; and gender, age, ethnicity, race, and income entered as covariates. Prior research suggests a usual care quit rate of 10% based on the 11% quit rate finding for the printed version of the *Guía Para Dejar de Fumar* in the Muñoz et al [13] study and missing=smoking rates ranging from 6.0% to 14.5% in our online studies [16,18,20]. We have estimated the quit rate of the improved, user-centered Web app at 20% based on the 23% quit rate found for the combined intervention in a later Muñoz et al study [13] and the best estimates of our online website, which yielded a missing=smoking rate of 20.2% for Spanish speakers and 21% for English speakers [16]. We estimate an increase of 10% (ie, improved website will result in 20% quit rate compared to a 10% quit rate for the baseline app), which results in an odds ratio of 2.25. This odds ratio was used as the effect size estimate in an a priori power analysis that yielded a total sample size of N=550 (225 per site version) to achieve power of .80.

We estimate needing a minimum of 800 participants to detect small effect sizes in utilization and 550 to detect clinically significant differences in quit rates. We therefore propose to recruit 800 participants via the open online recruitment process (to reach the minimum sample size estimated in our power analysis) and an additional 200 SFHN participants to increase our sample size and carry out secondary subgroup analyses to determine whether utilization and quit rates for the SFHN patients appear similar to those recruited outside the SFHN.

## Results

The project was funded in July 2015. Enrollment is currently underway and is expected to be finished by 2018. The first results are expected to be submitted for publication in 2019.

## Discussion

### Dissemination Plan

**Figure 2:**
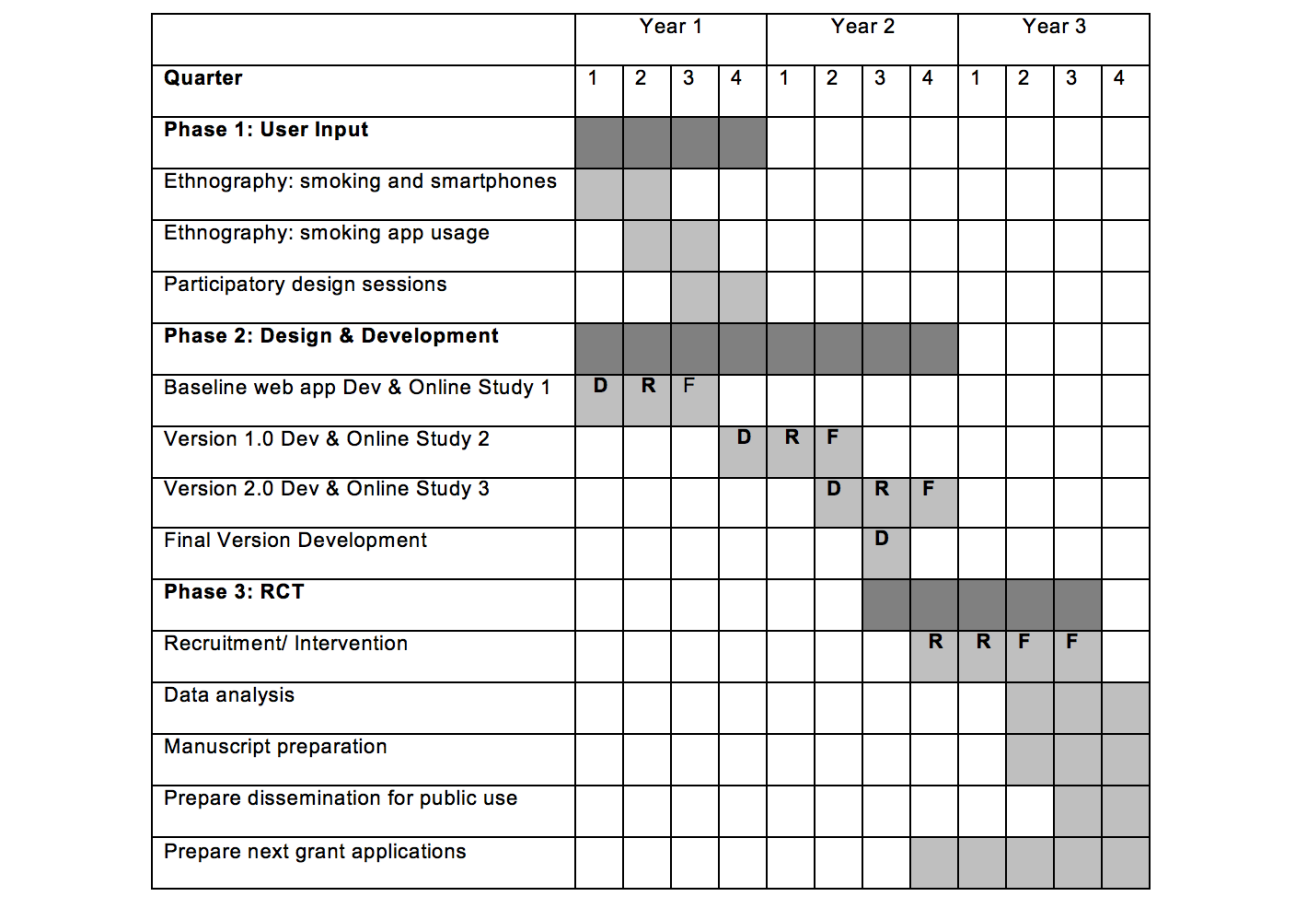
Timeline. Note: D=Development, R=Recruitment, F=Follow-ups

Our research team is committed to the dissemination of our research products. We disseminated the findings supported by our three earlier TRDRP grants (7RT-0057, 10RT-0326, and 13RT-0050) to the tobacco research community via 19 published articles and many additional posters and papers presented at local, national, and international venues. We plan to do the same with the research findings stemming from this project. In addition we actively sought continuous funding to extend the reach and duration of the intervention resulting from our three previous TRDRP grants. Since we were first funded in 1998, we have documented 347,000 visitors from more than 200 countries and territories and 52,268 consented participants in several online smoking cessation trials. For the participant preference trials, we made the *Guía Para Dejar de fumar* stop-smoking guide available on our home page to all who visited the site, whether or not they chose to proceed beyond the landing page. They were allowed to click on the link to the guide to download it without registering or having to pay for it. More than 258,340 visitors were given access to the guide. And, if our best estimates of quit rates are accurate, 20% of the 52,268 who registered, consented, and used our online interventions may have quit smoking, a total of more than 10,000 individuals. The research site remained active for 16 years after our initial grant was awarded. We intend to make the San Francisco Stop Smoking Web app resulting from the proposed project available to smokers worldwide after the project period terminates. For a complete timeline of the research study see Figure 2.

### Human Subjects and Ethical Considerations

The project received approval from the institutional review board at Palo Alto University in January 2016. Many of the study materials have been widely distributed and are well validated, and we do not anticipate any negative physical, mental, emotional, legal, or social consequences stemming from this study that are beyond what an individual would encounter in daily life. The Web app is presented to potential participants as a self-help automated research intervention (similar to a self-help book) and as neither counseling nor therapy. Therefore, use of the app does not involve a therapeutic contract. Participants are not compensated for interacting with the app, but they may receive indirect benefits such as a reduction in smoking behavior. Participants may withdraw from the study at any time and may refuse to answer any questions. During the study enrollment process, participants are informed that they may refuse to have their data used for official research reports but may still use the app. Their data will still be collected and used solely for the research team's ongoing Web app development.

All participants are informed that under no circumstances will their information be shared with their doctor or anyone outside the research team without their consent and that any published data will be aggregated and deidentified. All components of the Web application are provided in English and Spanish, and the previously validated stop-smoking guide has been shortened and simplified to a sixth-grade reading level. The project aims to sample equal numbers of male and female participants as well as English and Spanish speakers.

The first year of the current protocol has been registered at ClinicalTrials.gov Protocol Registration and Results System [NCT02666482].

### Conclusion

This study is intended to increase the range of utilization and effectiveness of a smoking cessation Web app beyond well-educated users. English- and Spanish-speaking low-income primary care patients of a public-sector health network will be asked to contribute to the development of the new Web app via ethnographic interviews and focus groups. After several iterations of the Web app, an RCT will be conducted to determine whether the final version of the Web app is superior to the baseline version in terms of acceptance, utilization, and higher abstinence rates.

## Abbreviations

ITEM: individually timed educational message
NCI: National Cancer Institute
R3: rapid, responsive, relevant
TC: Taking Control of Your Life
TRDRP: Tobacco-Related Disease Research Program
RCT: randomized controlled trial
SES: socioeconomic status
SFHN: San Francisco Health Network

## Appendix

Multimedia Appendix 1 Reviews of TRDRP grant.
