# Acknowledgements Correction of: Using Behavioral Intervention Technologies to Help Low-Income and Latino Smokers Quit: Protocol of a Randomized Controlled Trial

**DOI:** 10.2196/resprot.6635

**Published:** 2016-09-23

**Authors:** Ricardo F Muñoz, Eduardo Liniers Bunge, Alinne, Z Barrera, Robert, E Wickham, Jessica Lee

**Affiliations:** ^1^ Palo Alto University Palo Alto, CA United States

The authors of “Using Behavioral Intervention Technologies to Help Low-Income and Latino Smokers Quit: Protocol of a Randomized Controlled Trial” (JMIR Res Protoc 2016;5(2):e127) would like to change the Acknowledgements section of their paper to the following: 

“This project was partially supported by funds provided by The Regents of the University of California, Tobacco-Related Diseases Research Program, Grant Number No. 24RT-0027. The opinions, findings, and conclusions herein are those of the authors and not necessarily represent those of The Regents of the University of California, or any of its programs. 

Programming and development of the web app for this project is being carried out by the Center for Behavioral Intervention Technologies at Northwestern University Feinberg School of Medicine.”

The originally published acknowledgement has only the first sentence. 

This correction has been made in the online version of the paper on the JMIR Research Protocols website on September 23, 2016, together with publishing this corrigendum.

A correction notice has been sent to PubMed, and the publication was resubmitted to Pubmed Central and other full-text repositories.

