# Supplementary material for: Using Behavioral Intervention Technologies to Help Low-Income and Latino Smokers Quit: Protocol of a Randomized Controlled Trial
Source: JMIR Res Protoc. 2016 Jun 14;5(2):e127. doi: 10.2196/resprot.5355 (PMC4925932; doi:10.2196/resprot.5355)
Supplement: Multimedia Appendix 1 [file resprot_v5i2e127_app1.pdf]

TOBACCO-RELATED DISEASE RESEARCH PROGRAM
SUMMARY STATEMENT
Research Project Award (RT)

PI Name: Ricardo Muñoz
Title: Using Technology to help low-income and Latino smokers quit

REVIEWER 1

SUMMARY OF AIMS AND METHODS (PRIMARY & SECONDARY REVIEWERS ONLY)

Based on the several TRDRP projects funded previously and the successes of the www.stopsmoking.ucsf.edu, this study aims to revise and create smoking cessation digital tools (web app with text messaging components) to address the needs of low income smokers and in particular Spanish and English speaking Latino smokers. The project will engage low income and Latino smokers as well as their health care providers in the San Francisco Health Network (SFHN) in the process of creating and testing this app the project will identity effective ways of reaching and encouraging them to use it and evaluating the results, and deploying this app within the SFHN, CA and through a randomized control trial. The study aims to validate the use of internet tested technologies and mobile devices and further demonstrate how these can serve as cost effective, sustainable universal health care resources.

The International Internet Interventions for Health at Palo Alto University will partner with the Center for Behavioral Intervention Technologies (CBIT) at Northwestern University to iteratively develop the various versions of this app created through a human centered approach. Self help automated interventions have proven low cost, efficacious and long lasting. The researcher proposes a user-centered approach to integrate the actual patterns of usage of these types of devices by the target population utilizing the Design Thinking Process of five steps: Empathize, Define, Ideate, Prototype and Test. The proposal details the various steps and the Flow of their research. Furthermore, they will carry out this study inspired by Riley making it "rapid, responsive, relevant (R3) research. In addition they have planned a Six face-to-face field studies which will develop and test the app using qualitative and quantitative data, and four online studies to provide continuous quality improvement data. Ultimately, the study will evaluate the quit rates of those using the device online with 800 users. Previous TRDPR grants yielded 19 published articles and many poster and paper presentations, therefore this follow up research has the potential to impact thousands of smokers not just in CA but worldwide.

CRITIQUE:

Significance:

Yes, this study will provide a tangible app that can be used to reach low income smokers and Spanish and English speaking Latinos, both men and women. By showing how to actually engage the target population in creating usable technology based tools this study will serve as an example not just for tobacco cessation interventions but to address management of other chronic diseases. Through a three year phased approach that systematically tests the various versions of this digital tool, the methods and phasing may impact other innovative approaches to address health disparities. Utilizing human centered approaches will validate again the importance of engaging patients within a health care provider network such as SFHN and partnerships with technology centered researchers.

Approach:

The project has been well reasoned and is based on lessons learned after16 years of research in this field. The three year time framework will assure that the phases have ample time to not just work with the smokers, shadowing them and basing the development of this new app on why, when, how they use their smart phones but will engage them in group discussions to enrich the process. Having an English language and a Spanish language approach, the researchers are allowing for possible variations on the design and features of the app. I did not see any questioning on what would happen if they can't recruit and/or if there are problems with the design of the app. There is no "plan B" contemplated, though the three year framework allows for time to adjust in accordance to possible obstacles.

Innovation:

Yes, this is an innovative project. It is true that smart phones are being used already to have text messages reminding patients to take medication, to attend classes, to assure that they show up for medical visits, it is innovative to create a smoking cessation tool in itself that can be downloadable for free and based on how smokers use their phones already. This researcher and his team has been at the forefront of web based smoking cessation that has reach 347,000 users worldwide. What has been missing is an app or technology that reaches low income Latinos effectively. The partnership with Northwestern can help others think about innovative partnership between health care provider networks and technology companies that are patient centered.

Investigator:

Dr. Munoz has proven that he has the capability to carry out multiple research projects and produce not just usable tools but publish and disseminate the findings. The work proposed is in keeping with his interest and he has engaged other experienced researchers such as Dr. Eliseo Perez Stable in this proposal and is teaming with other researchers who know their fields.

Environment:

Yes, Dr. Munoz has successfully completed several TRDRP funded projects. He has the years of experience with the SFHN to assure that the partnership with them is solid. In addition, the website he created has continued for over 16 years regardless of whether it is funded or not, thereby proving that he can build sustainable methodologies and systems that can live after the grant period ends. There are letters of support and also detailed descriptions of the contracts that he intends to use to build the app.

RESEARCH RISKS:

Protection of Human Subjects from Research Risk

Acceptable. The IRB for this project is pending, but with the experience behind him and the guarantees the team has it is likely to be successful.

Inclusion of Women, Minorities and Children in Research:

This research adequately reaches low income men and women who are Latinos. He does not address the subgroups since the goal is to make a universal tool based on the language of choice, English and Spanish. The recruitment and retention of smokers in the developmental phase may be harder than planned even if the numbers are small at first. Plan B may be necessary even as the numbers of low income Latino smokers in the SFHN is high. But the research plan has various ways in which to address recruitment in other phases of the project such as online recruitment and retention.

OVERALL EVALUATION / PRIMARY REASON FOR RECOMMENDED MERIT RATING:

This is an outstanding proposal based on lessons learned in other project and has excellent chances of success. It may bring forth cultural differences between men and women and/or Spanish/English smokers and effective approaches. Strengths: It is innovative, has an actual usable product as a goal, uses multiples methodologies, it is based on ample experience in this field though it is trying out new usages for hand held devices. It is well planned, has enough patient engagement through the various phases, has measures of success, and good partnerships. Weaknesses: It is not clear whether there will be a link between the use of the app and the use of NRT at any point, though we know that Latinos tends not to use NRT as much as others. Since the product will be patient centered, it is hard to know if the result will be a completely self-help automated intervention and/or if it will have one or more guided interventions, and/or peer interventions and how the use of gaming, winning points and/or badges will work and who will "staff" these interventions once the app is live. The current website needs updating, but it is unclear if funds will be used to upgrade it. It is assumed that these two interventions will be linked (web/app), but the funding of the continuity of service is unclear and/or if the SFHN will direct its smokers to the website and/or the app continuously, or the system will engage smokers through the website. Something that is not addressed is the COST to the end user. Smartphones sometimes have charges for the time that users spend "on texting" or "on line". Will this app (even if it has limited "lessons") cost the users more than they are willing to pay in terms of added minutes/cost?

BUDGET RECOMMENDATIONS (PRIMARY & SECONDARY REVIEWERS ONLY):

The project should be funded as presented.

REVIEWER 2

SUMMARY OF AIMS AND METHODS (PRIMARY & SECONDARY REVIEWERS ONLY)

This study seeks to a) develop a human-centered bilingual smoking cessation web app; b) improve dissemination strategies through the identification of effective ways of reaching and encouraging low-income English- and Spanish-speaking smokers to use the digital smoking cessation interventions to be developed; c) Evaluate the effectiveness of the resulting stop smoking web app. The study methods are based on the "rapid, responsive, relevant research" approach (Riley, 2013) and will include iterative development of digital interventions for smoking cessation in Spanish and English smokers using input from patients as well as a five-step cyclic design. In addition, the study will include ethnographic semi-structured interviews, which will guide the development of

recruitment and dissemination strategies. Lastly, the proposal will include an RCT testing the effectiveness of the web app.

CRITIQUE:

Significance:

Low-income and Spanish speaking populations are an understudied health disparities population with respect to smoking, treatment access and smoking related diseases. Developing web-based applications for smoking cessation is desperately needed and this team of investigators is well positioned to make a continued contribution to this area. Findings can both inform future efforts with this population and also have a direct impact on the problem. Furthermore, the application of a human centered model could also serve as a catalyst for future studies and efforts. Overall, the proposed study is highly significant. One issue was noted: The proposal emphasizes the generalizability and dissemination of the web app and findings on a global scale particularly in low-income countries. However, the approach and proposal focuses heavily on the US (obviously a high income nation). Therefore, the issue of generalizability to low income nations is questionable and this issue takes away from the study's significance.

Approach:

The approach is rigorous and well articulated. There were no major design flaws noted.

Innovation:

The investigators have conducted similar work in the past and thus, there could have been a greater level of innovation based on previous findings or new developments in the field. For example, integrating elements of new and emerging tobacco products or including a social media component.

Investigator:

This is a highly qualified group of investigators with a strong track record of grants and publications. There are no weaknesses in this area.

Environment:

The extent of research funding and infrastructure is not entirely clear. Given the relatively new and clinical nature of the Department and institution (as compared to Dr. Munoz's previous institution and defined in the application) it seems important to discuss this to a greater extent.

RESEARCH RISKS:

Protection of Human Subjects from Research Risk

Acceptable

Inclusion of Women, Minorities and Children in Research:

Plans are acceptable

OVERALL EVALUATION / PRIMARY REASON FOR RECOMMENDED MERIT RATING:

This was a very strong application from a well-qualified and established team of investigators. The only significant weakness was a lack of innovation when considering a) the web is a highly evolving and innovative area of focus b) the investigators have done work in this area and could have derived more innovative elements based on previous findings.

BUDGET RECOMMENDATIONS (PRIMARY & SECONDARY REVIEWERS ONLY):

There are a number of faculty investigators with minor effort on this grant. This raises two concerns: 1) The likelihood of role overlap and the possible need for removing investigators. 2) The need for one senior investigator that will contribute a greater amount of effort.

REVIEWER 3

SUMMARY OF AIMS AND METHODS (PRIMARY & SECONDARY REVIEWERS ONLY)

Not applicable given this is the third reviewer.

CRITIQUE:

Significance:

The study addresses several important issues, including the disproportionate impact of smoking on low-income populations, and lower rates of utilization of cessation aids among Spanish-speaking smokers. The proposal makes a compelling case for the advantages of low-cost automated/digital self-help interventions (relative to more typical “consumable interventions”), and favors an "agile development process" to maintain a current look-and-feel. The research team has previously demonstrated follow-through in the development and dissemination of novel interventions, and digital self-help interventions hold promise for increasing reach and utilization of services among underrepresented and low-income populations.

Approach:

The conceptual framework (based on a user-centered approach to innovation) is very well developed, integrated, and appropriate to the aims. The use of ethnographic semi-structured interviews to inform both development and dissemination strategies is compelling.

For a fairly complex design, the methods were clearly described and well-reasoned, including both ethnographic/field test studies (e.g., smoking, cessation attempts, smartphone use, app use, participatory design, usability testing) and online studies (e.g., usual care web app vs. Versions 1/2) that will employ a mix of qualitative and quantitative approaches.

The data analytic approach and target sample sizes (N = 800 via online recruitment + 200 SFHN participants) were well-reasoned and specific to the respective study outcomes (e.g., n = 800 to detect small effects in utilization, n = 550 to detect differences in quit rates, and additional participants to conduct subgroup analyses).

The proposal acknowledges potential problem areas (e.g., highly educated people may be more likely to use digital interventions) and draws upon emerging research to conclude that these issues may have less to do with access and more to do with interventions not taking into account the characteristics of smokers with lower incomes/education.

Innovation:

There are several innovative aspects to the proposal, including: a focus on underrepresented and low-income populations; challenges to existing paradigms that tend to rely on costly interventions that can only be used once; the application of novel user-centered design processes (e.g., design thinking); and intention to take an "agile" development approach to maintain the utility of newly developed tools and technologies.

Investigator:

The investigative team is very strong with requisite expertise in the development, testing, and dissemination of novel interventions (e.g., for depression, substance use, smoking cessation) that have been delivered in both Spanish and English, both in person and via internet-based platforms.

Environment:

The scientific environment (especially the history of useful collaboration with SFHN) will likely contribute to the probability of success. The proposal benefits from unique features of the target population (e.g., low-income smokers), and there is good evidence of institutional support.

RESEARCH RISKS:

Protection of Human Subjects from Research Risk

Acceptable

Inclusion of Women, Minorities and Children in Research:

Acceptable

OVERALL EVALUATION / PRIMARY REASON FOR RECOMMENDED MERIT RATING:

Strengths include a focus on underrepresented and low-income populations; application of a user-centered development processes; development/testing of low-cost digital self-help interventions; the employment of both qualitative and quantitative methods; a strong and experienced investigative team; and a scientific environment that should contribute to the probability of success.
